# *In vitro* and *in vivo* activity of omadacycline against *Mycobacterium tuberculosis*

**DOI:** 10.3389/fmicb.2026.1789810

**Published:** 2026-06-09

**Authors:** Shahebraj Khan, Duc Nguyen, Dejan Nikolic, Baojie Wan, Akil Hossain, Mallique Qader, Sanghyun Cho, Scott G. Franzblau, Gauri S. Shetye

**Affiliations:** Department of Pharmaceutical Sciences, Retzky College of Pharmacy, Institute for Tuberculosis Research, University of Illinois Chicago, Chicago, IL, United States

**Keywords:** bedaquiline, linezolid, *Mycobacterium tuberculosis*, omadacycline, pretomanid, protein synthesis inhibitors, TB relapse mice model, tetracyclines

## Abstract

The bedaquiline (B), pretomanid (Pa), and linezolid (L) (BPaL) regimen is highly effective for drug-resistant tuberculosis (TB) but is limited by linezolid-associated toxicities, including myelosuppression and peripheral neuropathy. We evaluated omadacycline as a representative tetracycline-class protein synthesis inhibitor (PSI) to assess whether it can contribute functional activity within a BPa backbone. Omadacycline exhibited a minimum inhibitory concentration (MIC) of 2–4 μg/mL against *M. tuberculosis* H37Rv and 10–48 μg/mL against representatives of four global clades. Daily media replacement reduced the MIC against H37Rv to 0.29 μg/mL, suggesting antibiotic instability may underestimate *in vitro* potency. *In vivo*, subcutaneous administration of omadacycline (15 mg/kg) in BALB/c mice, yielding plasma exposures within the range of those reported for the approved 300-mg oral human dose, led to a 0.7-log_10_ reduction in lung colony-forming units (CFU) in both acute and chronic TB models. In combination therapy, 10-week treatment with BPa plus either linezolid (BPaL) or omadacycline (BPaO) further reduced lung CFU by ∼1 log_10_ relative to BPa alone, demonstrating the contribution of a PSI to regimen efficacy. Notably, BPaO and BPaL reduced relapse rates after 10 weeks of treatment to 43 and 23%, respectively, compared to 100% relapse in the BPa-only group. Together, these findings provide proof-of-concept evidence that omadacycline has *in vivo* antitubercular activity, achieves clinically relevant exposures, and can contribute functional PSI activity in combination regimens however, these results do not establish clinical equivalence with linezolid and support further evaluation of tetracycline-class agents in TB therapy.

## 1 Introduction

Tuberculosis (TB) remains a leading cause of death from infectious disease worldwide, with the emergence of multidrug-resistant (MDR) and extensively drug-resistant (XDR) strains posing a major threat to global control efforts (Sazali et al., 2023). Recent advances in TB drug development have led to the introduction of novel regimens that significantly shorten treatment duration and improve outcomes for drug-resistant TB (Conradie et al., 2020; Nyang’wa et al., 2024). Among these, the all-oral combination of bedaquiline (B), pretomanid (Pa), and linezolid (L) (BPaL) has demonstrated high efficacy in clinical trials and represents a major therapeutic milestone (Conradie et al., 2022). However, the widespread utility of BPaL is constrained by the dose- and duration-limiting toxicities associated with linezolid, including peripheral neuropathy and myelosuppression,(Vanino et al., 2023; Brandariz-Núñez et al., 2019; Gerson et al., 2002) highlighting the need for alternative protein synthesis inhibitors (PSIs) that can preserve regimen efficacy while improving tolerability (Ali et al., 2024).

PSIs play a critical role in modern TB combination therapy by contributing to both early bacteriostatic activity and sterilizing efficacy (Srivastava et al., 2017; Dietze et al., 2008). While linezolid has proven effective in this role, its toxicity profile has prompted interest in identifying safer PSI alternatives. Tetracyclines are a well-established class of broad-spectrum PSIs with favorable intracellular activity and pulmonary distribution (Gengenbacher et al., 2020; Walker et al., 2012; Gelhaus et al., 2013; Nelson and Levy, 2011; Pearson et al., 2025). Historically, earlier-generation tetracyclines demonstrated limited efficacy against *Mycobacterium tuberculosis*, likely due to suboptimal *in vivo* exposure rather than intrinsic inactivity (Duggar, 1948; Steenken and Wolinsky, 1950; Steenken and Wolinsky, 1949). Advances in tetracycline chemistry have yielded newer derivatives with improved stability, tissue penetration, and pharmacokinetic properties, prompting renewed interest in their potential application in TB therapy (LaPlante et al., 2022; Liu et al., 2025; Gallagher, 2019; Deshpande et al., 2022).

Omadacycline is a next-generation aminomethylcycline approved for the treatment of community-acquired bacterial pneumonia and acute bacterial skin and skin structure infections (Burgos and Rodvold, 2019; Durães and Sousa, 2019; Bidell et al., 2020). It exhibits activity against several clinically relevant intracellular respiratory pathogens,(Dubois et al., 2020; Wang et al., 2024) achieves therapeutically relevant concentrations in lung tissue,(Sun et al., 2025; Chapagain et al., 2022) and has an established safety profile that supports extended dosing relative to existing protein synthesis inhibitors (Mingora et al., 2023; Opal et al., 2019; Siddiqa et al., 2023). Preclinical pharmacodynamic studies, including hollow fiber infection models, have demonstrated antitubercular activity at exposures achievable with the approved 300-mg oral human dose, providing a rationale for evaluating omadacycline *in vivo* (Singh et al., 2024; Singh et al., 2023). Together, these characteristics position omadacycline as a candidate PSI for exploration within combination TB regimens.

In this study, we evaluated the *in vitro* activity, pharmacokinetics, and *in vivo* efficacy of omadacycline against *M. tuberculosis* using established murine models of TB infection. The primary objective of this study was to establish proof of concept for tetracycline-class PSIs as contributors to TB combination regimens, using omadacycline as a representative agent. By addressing this question, we aim to inform the development of PSI components in future TB combination regimens.

## 2 Results

### 2.1 *In vitro* activity

Omadacycline exhibited activity against *M. tuberculosis* strains H37Rv and Erdman, with a minimum inhibitory concentration (MIC) of 2.8 and 6.06 μg/mL, respectively (Table 1). The MICs of doxycycline and minocycline against H37Rv were 11.2 and 2.2 μg/mL, respectively. Activity of omadacycline against five *M. tuberculosis* clinical isolates representing four lineages (Filliol et al., 2002) ranged from 10 to 43 μg/mL (Table 1). In contrast to omadacycline, less variation in activity was observed for doxycycline and minocycline against global clades. The MIC fold difference between doxycycline and omadacycline, as well as between minocycline and omadacycline, across H37Rv and the five major *M. tuberculosis* clades (Filliol et al., 2002) ranged from 0.25- to 5-fold (median: 3) and 1.3- to 6.6-fold (median: 5), respectively. To provide broader context for tetracycline-class activity across mycobacteria, omadacycline was also evaluated against selected non-tuberculous mycobacteria (NTM), where it demonstrated activity against *M. abscessus*, *M. chelonae*, and *M. marinum* (Table 1) with a MIC range of 4–22 μg/mL (Table 1).

**TABLE 1:** MICs of omadacycline, doxycycline, and minocycline against *M. tuberculosis* H37Rv, *M. abscessus*, *M. chelonae*, and *M. marinum*.

	MICs (μg/mL)				
Mycobacterial strains	Omadacycline^#^	Doxycycline	Minocycline	Rifampin^#^	Bedaquiline^#^
*M. tuberculosis* (H37Rv)	2.80	11.20	2.17	0.05	0.04
Erdman	6.06	8.59	1.44	0.03	0.06
1:X003899	43.15	28.05	11.83	0.04	0.09
2:X004244	8.68	1.73	1.41	0.01	0.03
2:X004439	21.12	5.41	4.21	0.01	0.04
3:X001354	9.88	2.91	2.00	0.01	0.09
4:X005282	41.01	17.66	6.20	0.01	0.06
Nontuberculous Mycobacteria					
*M. abscessus*	3.80	> 50	> 50	> 6	0.14
*M. chelonae*	1.50	12.41	5.80	3.37	0.07
*M. marinum*	21.50	4.90	3.10	0.29	0.03

^#^ MICs are average values from three biological replicates.

Given the reported instability of omadacycline under prolonged incubation conditions, MIC testing was repeated with daily drug-media replacement during the 7-day incubation period. Under these conditions, the MIC of omadacycline decreased 14-fold compared to assays in which drug was not replenished (Table 2). In contrast, the MIC of rifampin remained unchanged with daily replenishment.

**TABLE 2:** MIC of omadacycline and rifampin with drug not replenished versus drug replenished daily.

	MIC^#^ (μg/mL) against *M. tuberculosis*	
Condition	Omadacycline	Rifampin
Drug not replenished	4.1	0.05
Drug replenished daily	0.29	0.02

^#^MICs are average of two biological replicates.

### 2.2 Pharmacokinetics of omadacycline in mice

**FIGURE 1:**
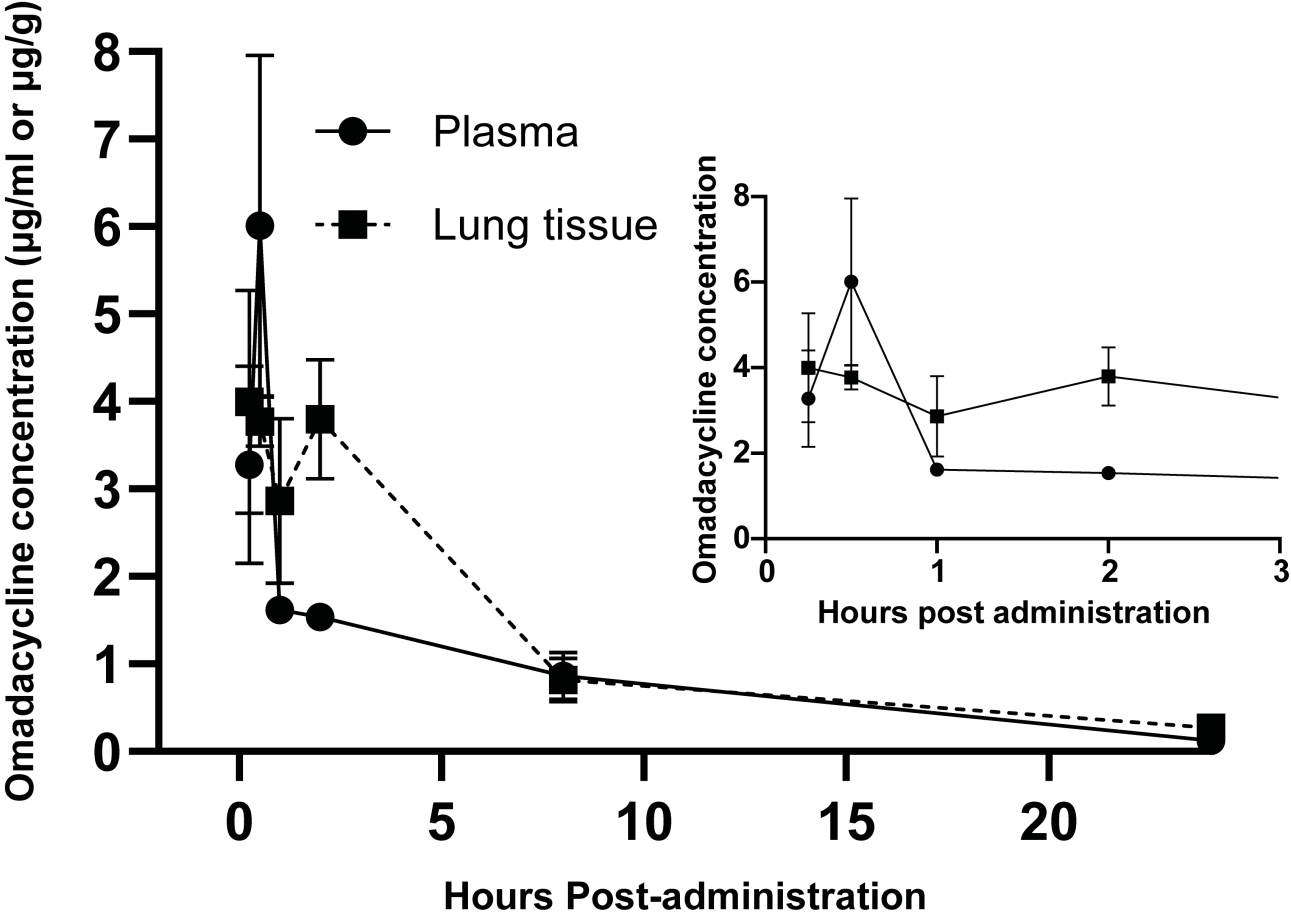
Plasma and lung exposures of omadacycline in mice. Mean plasma (μg/mL) and lung (μg/g) concentrations of omadacycline were measured over 24 h after the fourth dose following once-daily subcutaneous administration of 15 mg/kg for 4 days in BALB/c mice (*n* = 3 per time point). Plasma (●) and lung (■) concentration–time profiles demonstrate sustained systemic and pulmonary exposure. Inset: expanded view of the early time points (0–2 h) highlighting peak plasma and lung concentrations.

The pharmacokinetics of omadacycline were evaluated in female BALB/c mice following once-daily subcutaneous administration of 15 mg/kg for 4 days. Omadacycline appeared rapidly in systemic circulation, with measurable plasma and lung concentrations detected as early as 0.25 h (Figure 1). Mean plasma concentrations peaked at approximately 6.01 μg/mL at 0.5 h, followed by a log-linear decline with an apparent terminal half-life of approximately 5.95 h. Noncompartmental analysis of plasma concentrations yielded an AUC_0–24_ of 19.73 μg⋅h/mL (Table 3). Lung exposure was slightly higher and more sustained than plasma, with a lung AUC_0–24_ of 28.4 μg⋅h/g. Lung concentrations ranged from approximately 4.0 μg/g at early time points to 0.27 μg/g at 24 h following the final dose (Table 3). Notably, lung concentrations at 24 h approached the adjusted MIC (∼0.29 μg/mL, accounting for media instability). Although lung concentrations are expressed per gram of tissue, these data provide pharmacokinetic support for evaluating omadacycline efficacy in *M. tuberculosis*–infected mice.

**TABLE 3:** Pharmacokinetic parameters of omadacycline in plasma and lung following 15 mg/kg subcutaneous dosing in BALB/c mice.

Parameter	Plasma	Lung
Cmax	6.01 μg/mL	4.0 μg/g
Tmax	0.5 h	0.25–2 h
AUCτ (0–24 h)	19.73 μg⋅h/mL	28.39 μg⋅h/g
t½, app	5.95 h	-
n	3	3

Lung AUC was calculated using mean concentration–time data and is expressed per gram of tissue; terminal elimination parameters were not estimated for lung.

### 2.3 *In vivo* efficacy

Omadacycline was evaluated as a monotherapy in both acute and chronic TB BALB/c mouse infection models, which predominantly develop cellular granulomas. Due to poor oral bioavailability in mice, omadacycline was administered subcutaneously at 15 mg/kg of body weight per day, yielding a free drug AUC_0–24_ comparable to that of humans receiving the current standard oral dose of 300 mg/day (Singh et al., 2024; Rimal et al., 2023).

In the acute model, after 3 weeks of treatment (5 consecutive days per week), omadacycline reduced the *M. tuberculosis* burden in the mice lungs by 0.7 log_10_ CFU compared to the untreated control (*p* = 0.0013). Minocycline showed a 0.3 log_10_ reduction (*p* = 0.0009), while doxycycline, administered at the same dose, demonstrated no significant efficacy (Table 4). In the chronic model, after 4 weeks of treatment (5 days a week), omadacycline and linezolid reduced the bacterial burden by 0.7 log_10_ CFU (*p* = 0.0007) and 1.8 log_10_ CFU (*p* = 0.0008), respectively (Table 5). Because minocycline and doxycycline were less active in the acute infection model, they were not evaluated in the generally less permissive chronic model. Rifampin was used as a positive control in both models.

**TABLE 4:** Lung CFU in BALB/c mice after 3 weeks of treatment with omadacycline and second-generation tetracyclines in the acute model.

Drugs	Dosage/route	Mean lung log_10_ CFU (± SD)			Log reduction
		T3	T10	T31	
Untreated	15 mg/kg (SC)	2.3 (± 0.3)	3.6 (± 0.17)	6.6 (± 0.13)	0.7**
Omadacycline				5.9 (± 0.16)	
Minocycline	50 mg/kg (PO)			6.3 (± 0.13)	0.3***
Doxycycline	50 mg/kg (PO)			6.6 (± 0.11)	0
Rifampin	15 mg/kg (PO)			3.9 (± 0.17)	2.7***

***p < 0.001,

**p < 0.01 compared to untreated group, *n* = 3 mice were euthanized at T3 to confirm pulmonary implantation of Mtb, and *n* = 5 mice were euthanized at T10 to establish baseline Mtb burden prior to treatment initiation. Treatment was initiated at T10 and continued for 3 weeks. All treatment groups at T31 consisted of *n* = 7 mice per group.

**TABLE 5:** Lung CFU counts in BALB/c mice after 4 weeks of treatment with omadacycline and linezolid in the chronic model.

Drugs	Dosage/route	Mean lung log_10_ CFU (± SD)			Log reduction
		T3	T31	T59	
Untreated	15 mg/kg (SC)	2.7 (± 0.21)	6.1 (± 0.14)	6.9 (± 0.11)	0.7***
Omadacycline				6.2 (± 0.20)	
Linezolid	100 mg/kg (PO)			5.1 (± 0.21)	1.8***
Rifampin	15 mg/kg (PO)			4.3 (± 0.11)	2.6***

***p < 0.001 compared to untreated group, *n* = 3 mice were euthanized at T3 to confirm pulmonary implantation of Mtb, and *n* = 5 mice were euthanized at T31 to establish baseline Mtb burden prior to treatment initiation. Treatment was initiated at T31 and continued for 4 weeks. All treatment groups at T59 consisted of *n* = 7 mice per group.

Omadacycline (O) was assessed in combination with bedaquiline (B) and pretomanid (Pa). The efficacy of the BPaO regimen was compared with the FDA-approved BPaL regimen (bedaquiline, pretomanid, and linezolid). The effectiveness of the BPaO, BPaL, and BPa regimens was tested in a high-dose chronic BALB/c mouse model. The BPa control arm was included to evaluate the impact of adding either PSI on treatment efficacy.

Neither linezolid nor omadacycline significantly enhanced the efficacy of BPa after 4 weeks of treatment (Table 6). However, after 10 weeks of treatment, both protein synthesis inhibitors (PSIs) contributed to an additional 1 log_10_ reduction in CFU compared to BPa alone (*p* = 0.01 for BPaO and *p* = 0.04 for BPaL). To assess the effect of omadacycline on the sterilizing potential of BPa, 15 mice from the BPa and BPaO groups, and 16 mice from the BPaL group, were maintained for an additional 3 months without further treatment. After this period, the mice were evaluated for culture positivity. All mice from the BPa treatment group remained culture-positive, while the addition of omadacycline or linezolid to BPa significantly reduced the percentage of culture-positive mice to 43 and 23%, respectively (Table 6).

**TABLE 6:** Impact of 4 and 10 weeks of treatment of omadacycline (15 mg/kg, SC) vs. linezolid (50 mg/kg, PO) when added to bedaquiline (25 mg/kg, PO) plus pretomanid (100 mg/kg, PO) in the BALB/c chronic infection model.

Regimen	Mean lung log_10_ CFU (± SD)				Mice relapsing after 10 weeks treatment^##^	
			Weeks of treatment		Proportion	% Relapse
	T3	T31	4	10		
Untreated	3.82 (± 0.05)	7.2 (± 0.17)	8.33 (± 0.26)	2.13 (± 0.26)	15/15	100
BPa			4.17 (± 0.26)			
BPaO			3.99 (± 0.14)	0.95 (± 0.85)*	7/16	43
BPaL			4.24 (± 0.13)	1.00 (± 0.70)*	3/13^#^	23

*n* = 5 mice were euthanized at weeks 4 and 10.

^##^ Relapse mice (*n* = 15) were held for 3 additional months after completing 10 weeks of treatment.

*P < 0.05 compared to BPa at 10 weeks.

^#^15 mice were held for relapse assessment in the BPaL group. CFU plates from 2 animals were contaminated and excluded from analysis; relapse proportion was therefore calculated from the remaining 13 evaluable mice.

## 3 Discussion

The primary objective of this study was not to position omadacycline as a clinical replacement for linezolid but to determine whether a tetracycline-class agent can demonstrate measurable *in vivo* activity against *M. tuberculosis* and contribute meaningfully to combination regimens. In this context, the observed reductions in lung bacterial burden and relapse rates provide proof-of-concept that omadacycline has functional antitubercular activity *in vivo*, despite modest MIC values under standard *in vitro* conditions.

The seemingly modest MIC of omadacycline against *M. tuberculosis*, as determined by standard methods, must be interpreted in the context of tetracycline instability. Tetracyclines are known to degrade under various temperature and media conditions (Schäfer and Knothe, 1978; Xuan et al., 2010; Zhang et al., 2012). Omadacycline shares this instability, with a half-life of ∼22 h in 7H9_OADC medium (Shankar et al., 2022; Singh et al., 2024). To address this, we replenished the drug daily during MIC testing, reducing the MIC 14-fold compared to conventional testing where fresh drug was not supplemented (Table 2). In contrast, rifampin, used as a control, showed no significant change in MIC values under either condition. Supporting these findings, earlier studies demonstrated a reduction in omadacycline’s MIC against multidrug-resistant *M. tuberculosis* (MDR-TB) from 16 to 4 μg/mL when only 50% of the drug was replenished daily in the medium (Shankar et al., 2022).

*M. tuberculosis* isolates collected worldwide can be grouped into six major phylogenetic lineages based on single nucleotide polymorphism (SNP) patterns (Filliol et al., 2002). Omadacycline exhibited variable activity across the five *M. tuberculosis* isolates tested, which represented four of these lineages. These lineages are defined by characteristic genetic polymorphisms that can influence drug susceptibility and resistance mechanisms through differences in efflux pump expression, cell wall permeability, and other lineage-associated traits (Castro et al., 2020; Shanmugam et al., 2022; Oppong et al., 2019). Assuming MIC reductions similar to those observed for H37Rv with drug replenishment, the estimated MIC for the least susceptible strain would remain around 3 μg/mL, which is below the pharmacodynamic threshold predicted for efficacy at the standard 300-mg oral human dose in hollow fiber model analyses (Singh et al., 2024). The observed activity against selected NTM species further supports the broader antimycobacterial potential of tetracycline-class agents, although the primary focus of this study remains *M. tuberculosis.* As with *M. tuberculosis*, interpretation of NTM MIC values should consider the impact of tetracycline instability under standard assay conditions. Given the prolonged incubation times required for slow-growing mycobacteria, this effect may be more pronounced than in rapidly growing species; however, this was not directly evaluated in the present study. Although minocycline exhibited lower MIC values *in vitro*, selection of omadacycline for further evaluation was based on its translational pharmacologic properties, including clinically established dosing, lower plasma protein binding, lung/tissue distribution, and pharmacologically favorable free-drug exposure characteristics, rather than on *in vitro* potency alone.

However, the *in vivo* efficacy studies were performed using the Erdman strain and may not fully capture the range of responses across diverse *M. tuberculosis* lineages, particularly those with higher apparent MICs. Interpretation of these MIC values should therefore consider the known instability of omadacycline under standard assay conditions, which may lead to overestimation of potency thresholds. Accordingly, the observed *in vivo* efficacy should be interpreted with caution when extrapolating across clinical strains with variable susceptibility. Although quantitative AUC/MIC targets have been defined primarily in non-mycobacterial models, the exposures achieved here are comparable to or exceed omadacycline AUC/MIC ratios associated with stasis and 1-log_10_ kill in murine pneumonia (Lepak et al., 2017) and hollow-fiber studies (Singh et al., 2024). These prior data support the interpretation that the 15 mg/kg regimen, provided pharmacologically active exposures in our *M. tuberculosis* model, while recognizing that the precise PK/PD target for *M. tuberculosis* remains to be defined and is informed by the present *in vivo* efficacy results.

In addition to preclinical PK/PD benchmarks, human pharmacokinetic data provide translational context for these findings. The plasma AUC_0–24_ achieved in mice following the 15 mg/kg subcutaneous regimen, after unit conversion, appear to fall within approximately two-fold of steady-state total plasma AUC_0–24_ values reported in adults receiving the approved 300-mg once-daily oral dose of omadacycline. This comparison serves as a qualitative exposure benchmark rather than a formal human dose prediction, indicating that the exposures associated with activity in the murine *M. tuberculosis* model are not grossly supratherapeutic relative to clinically attainable human exposures. The modest (∼1-log_10_) reduction in lung CFU observed under these conditions is consistent with the predominantly bacteriostatic activity of omadacycline and underscores the need for dedicated PK/PD studies to define exposure–response relationships and optimize combination regimens for tuberculosis. The PK analysis in this study was intended as an exploratory exposure assessment to provide translational context for the observed efficacy results rather than to establish definitive pharmacokinetic parameters. Future studies incorporating larger sample sizes, population PK approaches, protein binding evaluation, and lesion distribution analyses will be important to further define omadacycline PK/PD relationships in tuberculosis.

BALB/c mice were selected as a well-established model for initial efficacy and relapse assessment in tuberculosis, providing a controlled and reproducible platform for proof-of-concept evaluation of drug activity within combination regimens. However, this model develops predominantly non-necrotic, cellular granulomas that do not recapitulate the caseating pathology characteristic of human tuberculosis, which should be considered when interpreting the present findings. Within this model, the absence of necrotic lesions may influence drug distribution and could underestimate the activity of compounds that preferentially accumulate in caseous tissue. Future studies in models with more human-like pathology, such as C3HeB/FeJ mice, will be important to fully define the translational potential of omadacycline.

Omadacycline demonstrated modest but consistent activity across both experimental settings evaluated (Tables 4, 5). Despite differences in infection stage and bacterial physiology captured by these models, the magnitude of CFU reduction was comparable, supporting the conclusion that omadacycline exerts reproducible antitubercular activity under distinct disease conditions. The similar degree of bacterial reduction observed across models is consistent with a predominantly bacteriostatic mechanism of action and aligns with expectations for a protein synthesis inhibitor evaluated as monotherapy. Collectively, these findings indicate that omadacycline maintains measurable *in vivo* efficacy across multiple stages of tuberculosis infection, providing a rationale for its further evaluation in combination regimens rather than as a standalone agent.

Although BPaL regimen represents a major advance in drug-resistant TB therapy, the dose- and duration-limiting toxicities associated with linezolid, particularly peripheral neuropathy and myelosuppression, have motivated efforts to identify alternative protein synthesis inhibitors that preserve regimen efficacy while improving tolerability. Bedaquiline and pretomanid were retained as a clinically validated BPa backbone to enable controlled evaluation of PSI substitution. This design isolates the contribution of the PSI component without introducing additional variables from modifying multiple drugs. Accordingly, this study was not intended to propose a new clinical regimen or directly modify BPaL, but to establish proof-of-concept for tetracycline-class PSI activity within a clinically relevant context. Future studies evaluating tetracycline-class PSIs within other clinically relevant companion-drug backbones will be important to further define optimal regimen design. We therefore evaluated whether omadacycline could functionally contribute PSI activity within a BPa backbone. To investigate this, we compared the efficacy of three treatment regimens—BPa (bedaquiline and pretomanid), BPaL (bedaquiline, pretomanid, and linezolid), and BPaO (bedaquiline, pretomanid, and omadacycline)—in a chronic TB mouse model. At 4 weeks, bacterial burden reductions were similar across all regimens. However, after 10 weeks, BPaL and BPaO achieved approximately 1-log greater CFU reductions compared to BPa alone (Table 6), highlighting the contribution of a PSI to the regimen’s sterilizing activity. These results reinforce the importance of PSIs in enhancing treatment efficacy.

To evaluate long-term treatment outcomes, we assessed relapse after 10 weeks of therapy followed by a 3-month drug-free period. Treatment duration of 10 weeks was chosen to balance sufficient bacterial killing with the ability to detect differences in relapse rates between regimens; longer treatment might have driven bacterial counts to zero at treatment completion, obscuring the contribution of individual protein synthesis inhibitors to relapse prevention. Using this approach, all mice receiving BPa alone relapsed (100%), while relapse was significantly reduced in BPaL (23%) and, to a lesser extent, in BPaO (43%). Although relapse rates were lower with BPaL than BPaO, both regimens markedly outperformed BPa alone. The observed difference may reflect dose selection, pharmacokinetic properties, or intrinsic potency differences between protein synthesis inhibitors. While linezolid demonstrated numerically superior relapse suppression under the conditions tested, this study was not designed or powered to determine the equivalence of two regimens. Importantly, these findings do not establish clinical equivalence or interchangeability with linezolid, but rather support proof-of-concept for tetracycline-class protein synthesis inhibitors contributing to regimen activity. Further dose-optimization and PK/PD studies will be required to determine whether tetracycline-class agents can achieve relapse suppression comparable to linezolid while offering improved tolerability.

This is the first report demonstrating the *in vivo* efficacy of a tetracycline-class antibiotic against *M. tuberculosis* in a murine model. Previously, doxycycline demonstrated efficacy in a guinea pig model, which develops necrotic, caseating granulomas that closely resemble human TB pathology (Walker et al., 2012). In our study, however, doxycycline administered at 50 mg/kg in BALB/c mice failed to reduce lung bacterial burden. Inadequate drug exposure may have contributed to the lack of efficacy, as some PK data suggest that both our study and the Walker et al. guinea pig study likely fell short of achieving human-equivalent doxycycline exposures (Gelhaus et al., 2013; Heine et al., 2007). Therefore, the divergent outcomes are more likely driven by differences in lesion pathology rather than pharmacokinetics alone. BALB/c mice develop non-necrotic, cellular granulomas, which may limit drug penetration and reduce translational relevance (Leong et al., 2010). In contrast, guinea pigs and humans form caseating granulomas that can serve as drug reservoirs for lipophilic compounds (Silva Miranda et al., 2012). For example, rifampin accumulates to higher concentrations in caseum compared to cellular granulomas due to its ability to diffuse into necrotic tissue (Prideaux et al., 2015). Similarly, omadacycline’s higher lipophilicity may enhance its penetration into necrotic lesions, potentially surpassing older tetracyclines in efficacy (Lin et al., 2016). Thus, the modest activity observed in BALB/c mice may underestimate omadacycline’s true therapeutic potential. Future studies in our laboratory are underway to investigate omadacycline’s pharmacokinetics, lesion distribution, and immunomodulatory effects in the C3HeB/FeJ mouse model, which better recapitulates the caseating granulomas of human tuberculosis.

In summary, this work establishes proof of concept that tetracycline-class agents can function as protein synthesis inhibitors within clinically relevant TB combination regimens, using omadacycline as a representative agent with favorable pharmacokinetic properties. These findings support continued development of tetracycline-class PSIs in TB drug discovery and combination regimen design.

## 4 Materials and methods

### 4.1 Bacterial strains

*Mycobacterium tuberculosis* (Mtb) strain H37Rv (ATCC 27294), *M. chelonae* (ATCC 35752), *M. marinum* (ATCC 927), and *M. abscessus* (ATCC 19977) were studied. Additionally, Mtb isolates collected from various regions worldwide were categorized into six major single-nucleotide polymorphism (SNP) clusters (Filliol et al., 2002). Omadacycline was tested against isolates from three of these six clusters. Specifically, isolates X004439 and X004244 belonged to the East Asian lineage, X005282 to the Euro-American lineage, X003899 to Indo Oceanic lineage, and X001354 to the East African Indian lineage (Filliol et al., 2002).

### 4.2 MIC determination against Mtb, Mtb global clades and NTMs

All experiments with Mtb were conducted in a biosafety level 3 (BSL-3) laboratory. The minimum inhibitory concentration (MIC) against *M. tuberculosis*, its global clades, and nontuberculous mycobacteria (NTMs) was determined using the previously described Microplate Alamar Blue Assay (MABA) (Cho et al., 2015). Briefly, antibiotic stocks were prepared in dimethyl sulfoxide (DMSO) at 100 × the highest desired final concentration. A 2 μL aliquot of each antibiotic DMSO stock was transferred to an assay plate containing 100 μL of Middlebrook 7H12 medium [composed of 4.7 g 7H9 broth, 1 g casitone (Bacto), 5 g bovine serum albumin (BSA), 4 mg catalase, and 5.6 mg palmitic acid per liter of media]. For *M. chelonae* and *M. marinum*, Middlebrook 7H9 broth (BD) supplemented with 0.2% glycerol, 0.05% Tween and 10% oleic acid-albumin-dextrose-catalase (OADC) (BD Franklin Lakes, NJ) media was used. A twofold serial dilution of the antibiotic was performed nine times in the assay plate. The plates were then inoculated with bacterial cultures to achieve a final density of approximately 1 × 10^5^ CFU/mL. Assay plates were incubated at 37°C for 7 days for *M. tuberculosis* and its global clades. For *M. abscessus*, *M. chelonae*, and *M. marinum*, incubation times were 3, 3, and 5 days, respectively. After the incubation, a resazurin dye/Tween 80 mixture (0.6 mM resazurin dye and 12 μL of 20% Tween 80) was added to each well, and plates were further incubated for an additional 18–24 h at 37°C for Mtb and its global clades. For NTMs, plates were incubated with the resazurin dye/Tween 80 mixture for 4 h. Fluorescence was measured using a CLARIOstar (BMG LABTECH, Ortenberg, Germany) plate reader. The MIC was defined as the lowest concentration that resulted in a 90% reduction in fluorescence relative to the controls.

### 4.3 MIC determination with daily drug replenishment

To evaluate the impact of omadacycline instability during prolonged incubation, MIC testing with daily drug replenishment was performed in parallel with standard MABA assays. Instead of conducting the assay in 96-well plates, cultures were incubated in 1.5 mL microcentrifuge tubes with a final volume of 1 mL per tube. Two-fold serial dilutions of omadacycline were prepared in Middlebrook 7H12 medium as described above, and tubes were inoculated to a final density of approximately 1 × 10*5* CFU/mL. Tubes were incubated at 37°C. Every 24 h for 7 days, samples were centrifuged, the supernatant was carefully removed, and bacterial pellets were resuspended in fresh prewarmed medium containing the corresponding concentration of omadacycline. Rifampin was included as a control drug and was processed identically. After 7 days of incubation, 200 μL from each tube was transferred to a 96-well plate, and resazurin/Tween 80 solution was added as described above. Plates were incubated for 24 h at 37°C prior to fluorescence measurement. MIC values were defined as the lowest concentration resulting in ≥ 90% reduction in fluorescence relative to untreated controls.

### 4.4 Animals

Female BALB/c mice (8–10 weeks old; Charles River Laboratories, Wilmington, MA) were used for all *in vivo* studies. All animal procedures were approved by the University of Illinois Chicago Institutional Animal Care and Use Committee (IACUC; protocol #24-075) and were conducted in accordance with the Guide for the Care and Use of Laboratory Animals. Aerosol infection with *Mycobacterium tuberculosis* was performed in a certified BSL-3 facility. Following infection, mice were transferred to and maintained in an IBC- and IACUC-approved ABSL-2 containment facility specifically authorized for work with *M. tuberculosis*–infected mice. Housing and animal procedures in this facility were conducted in accordance with the Biosafety in Microbiological and Biomedical Laboratories (BMBL), 6th Edition (Centers for Disease Control and Prevention [CDC] and National Institutes of Health [NIH], 2020), which permits housing of *M. tuberculosis*–infected mice under ABSL-2 containment using BSL-3 work practices (p. 174). Chemotherapy administration and euthanasia were performed in the approved ABSL-2 facility using institutionally required BSL-3 work practices and appropriate personal protective equipment. Following necropsy, lungs were transported in sealed, leak-proof primary containment to a certified BSL-3 laboratory for tissue processing (homogenization) and CFU plating.

### 4.5 Pharmacokinetic studies

Female BALB/c mice (18–20 g) were administered omadacycline subcutaneously at a dose of 15 mg/kg once daily for four consecutive days. Following the fourth dose, blood and lung samples were collected at 0.25, 0.5, 1, 2, 8, and 24 h post-dose (*n* = 3 per time point). Blood samples were centrifuged to obtain plasma, and lung tissues were harvested, weighed, and homogenized prior to drug quantification. Omadacycline concentrations in plasma and lung homogenates were determined using LC-MS/MS following a validated analytical method with slight modifications (Chen et al., 2025). Samples were separated on a Waters (Milford, MA) XSELECT 2.0 × 50 mm C_18_ column, 3.5 μm particle size using a mobile phase consisting of water containing 5 mM ammonium formate + 0.1% formic acid (Solvent A) and acetonitrile (Solvent B) and the linear gradient from 6 to 80% B over 3 min at a flow rate of 0.3 mL/min. The column was thermostated at 40°C. Mass spectrometric data were acquired using positive ion electrospray ionization on a Shimadzu 8060 triple quadrupole mass spectrometer and the SRM transitions: 557/453 and 279/270 for the analyte and 458/352 for the internal standard minocycline. Noncompartmental pharmacokinetic analysis was performed using mean concentration–time data. Maximum observed concentration (Cmax) and time to Cmax (Tmax) were obtained directly from the observed data. The area under the concentration–time curve from 0 to 24 h (AUC_0–24_) was calculated using the linear trapezoidal method. The terminal elimination rate constant (λz) was estimated from the log-linear portion of the plasma concentration–time curve, and the terminal half-life (t½,app) was calculated as 0.693/λz. Lung AUC_0–24_ was calculated using mean concentration–time data and expressed per gram of tissue; terminal elimination parameters were not estimated for lung.

### 4.6 Aerosol infection

The *M. tuberculosis* Erdman strain (ATCC 35801) was used for aerosol infections of mice, and inoculum was prepared as previously described (Gao et al., 2015). Briefly, the bacteria were originally grown in 7H9 to generate low passage seed lots. Working stocks were generated by growing to mid-log phase, divided into 1.5 mL aliquots, and stored at -80°C until use. Mice were exposed to a low dose aerosol infection using a Glas-Col inhalation exposure system, as previously described, resulting in an average of 200–400 bacteria in the lungs after 3 days of exposure (T3).

### 4.7 Chemotherapy

Mice were randomized into treatment groups shortly after infection. In both the acute and chronic studies, three mice were euthanized at day 3 post-infection (T3) to confirm successful pulmonary implantation. In the acute model, an additional cohort of five mice was euthanized at day 10 post-infection (T10) to establish the baseline bacterial burden immediately prior to treatment initiation. Treatment was initiated at T10 and continued for 3 weeks. In the chronic model, infection was allowed to progress to a plateau phase. A separate cohort of five mice was euthanized at day 31 post-infection (T31) to establish baseline bacterial burden prior to treatment initiation. Treatment was initiated at T31 and continued for 4 weeks. All remaining treatment groups consisted of seven mice per group unless otherwise specified.

Drugs were prepared as previously described and administered once daily, 5 days per week, by gavage (Xu et al., 2019). The drug doses (in milligrams per kilogram of body weight, mg/kg) were the following: bedaquiline, 25; pretomanid, 100; linezolid, 100 (monotreatment) and 50 (combination regimen). The 50 mg/kg linezolid dose used in the combination relapse study was selected to align with lower linezolid exposure strategies used in subsequent preclinical and clinical BPaL investigations following recognition of dose-related toxicities (Lee et al., 2012; Mishra, 2021). Bedaquiline was formulated in an acidified 20% hydroxypropyl-β-cyclodextrin solution as previously described (Xu et al., 2019). Pretomanid was suspended in a cyclodextrin micelle (CM-2) formulation containing 10% hydroxypropyl-β-cyclodextrin (Sigma) and 10% lecithin (Tyagi et al., 2005). Linezolid was suspended in a solution composed of 5% polyethylene glycol 200 (PEG 200; Sigma) and 95% methylcellulose in distilled water (Tasneen et al., 2016). Omadacycline (15 mg/kg), minocycline (50 mg/kg) and doxycycline (50 mg/kg) were prepared in phosphate buffer saline. Omadacycline was administered subcutaneously to ensure consistent systemic exposure in mice, as oral bioavailability in rodents is low and subject to variability due to gastrointestinal chelation and dietary influences. Subcutaneous administration enabled more reliable PK/PD alignment with clinically relevant exposure targets. Injection sites were monitored throughout the treatment period. No ulceration, necrosis, or observable local tissue toxicity was noted, and animals maintained normal behavior and body weight during therapy. All other drugs were administered by oral gavage. All the drugs in combination were prepared and administered separately.

### 4.8 Assessment of treatment efficacy and relapse

Treatment efficacy was assessed based on lung CFU counts at the completion of 4 and 10 weeks of treatment and the proportion of mice relapsing with lung CFU detected upon sacrifice 3 months after completing 10 weeks of treatment. Serial dilutions of hundred microliter lung homogenates were plated on Middlebrook 7H11 agar plates enriched with 10% oleic acid-albumin-dextrose-catalase (OADC) (Becton Dickinson, Franklin Lakes, NJ) 200 U/mL of polymyxin B (Sigma) and 20 μg/mL of trimethoprim (Sigma). The plates were incubated for 3– 4 weeks at 37°C before determining the final CFU counts. Relapses were assessed by plating the entire lung homogenate to reduce the lower limit of CFU detection to one.

### 4.9 Statistical analysis

For CFU determination, duplicate plantings from each mouse were averaged prior to analysis. Lung bacterial burdens were expressed as log_10_ CFU per mouse. For acute and chronic monotherapy studies, comparisons were limited to pre-specified pairwise analyses between each treatment group and its corresponding control group at the same timepoint. For combination-regimen CFU endpoints of BPaO and BPaL groups were compared with the BPa group at the same timepoint (week 10). These comparisons were performed using unpaired two-tailed Student’s *t*-tests on log_10_-transformed CFU values in GraphPad Prism. Because comparisons were restricted to predefined treatment-versus-control analyses rather than all possible pairwise group comparisons, no additional multiplicity adjustment was applied.

## Data Availability

The original contributions presented in the study are included in the article/supplementary material, further inquiries can be directed to the corresponding author.
